# Elevated Levels of the Cancer Marker Neuron-Specific Enolase in a Patient With Coexisting Silicosis and Sarcoidosis

**DOI:** 10.7759/cureus.61130

**Published:** 2024-05-26

**Authors:** Khishigtogtokh Davaajav, Dolgormaa Dagva, Ichinnorov Dashtseren, Yoshimitsu Takahashi, Takeo Nakayama

**Affiliations:** 1 Health Informatics, Kyoto University, Graduate School of Medicine, Kyoto, JPN; 2 Forensic Medicine, National Forensic Agency of Mongolia, Ulaanbaatar, MNG; 3 Pulmonology and Allergology, Mongolian National University of Medical Sciences, School of Medicine, Ulaanbaatar, MNG

**Keywords:** cancer marker, occupational exposure to silica, lung cancers, pulmonary sarcoidosis, silicosis

## Abstract

In a periodical medical checkup, a 39-year-old Mongolian underground miner was diagnosed with silicosis based on chest radiography, computed tomography (CT), and work history. Chest radiography showed diffuse bilateral rounded nodules in both lung fields, with upper lobe dominance and large opacities in the right upper zone. Chest CT presented conglomerated massive changes in the right upper lobe and the coalescence of small nodules in the left upper lung. In the blood test, serum levels of the lung cancer marker neuron-specific enolase (NSE) were elevated (24.58 ng/mL). Carcinoembryonic antigen (CEA) and cytokeratin 19 fragment (CYFRA 21-1) levels were within the reference range. Subsequent to the suspicion of a tumour in the right upper lobe, a right upper lobectomy was performed. The histopathological examination of the lung specimen revealed the coalescence of numerous silica nodules, accompanied by indications of associated sarcoidosis. The histological features suggested the presence of two concurrent pathological processes: silicosis and sarcoidosis. This case demonstrated the combination of three clinical conditions diagnosed in one patient, including complicated silicosis associated with sarcoidosis and elevated serum NSE levels. This case report may serve as a foundation for future investigations exploring the potential of NSE as a marker for silicosis.

## Introduction

Silicosis, a disease with an extensive historical background, has been a persistent occupational health concern, primarily affecting workers who have been exposed to silica dust over an extended period [1]. Silica is the most abundant mineral in the world, comprising rock, sand, and soil. It causes silicosis and is associated with many other diseases, such as sarcoidosis, rheumatoid arthritis, and several autoimmune diseases [2].

In 1987, the International Agency for Research on Cancer (IARC) classified crystalline silica as a probable human lung carcinogen. Sarcoidosis is a chronic systemic granulomatous disease of unknown aetiology. In over 90% of patients with diagnosed sarcoidosis, mediastinal and hilar lymph nodes are affected [3].

Sarcoidosis is also associated with beryllium, radon dust inhalation, metal fume, wood dust, and silica dust [4-6]. Several epidemiological and case studies revealed the association between silica exposure and sarcoidosis, showing that sensitisation to silica could be involved in the underlying immunological mechanism. Whether silicosis and associated sarcoidosis are becoming a new phenotype of occupational lung disease is debatable [7,8].

Moore and McGregor first described the neuron-specific enolase (NSE) in 1965, a key enzyme in aerobic glycolysis. NSE is currently the most reliable tumour marker in the diagnosis, prognosis, and follow-up of small cell lung cancer (SCLC), even though increased levels of NSE have also been reported in non-SCLC (NSCLC). The level of NSE correlates with tumour burden, number of metastatic sites, and response to treatment [9-11].

In an effort to enhance the early detection of silicosis and expand diagnostic capabilities, particularly in situations where chest radiography is not accessible, alternative methods have been explored. Notably, research has indicated that serum NSE levels may serve as a valuable adjunct to chest radiography in determining the stage of silicosis and facilitating early diagnosis [12,13]. This paper reports the first case of silicosis associated with sarcoidosis diagnosed with an elevated serum NSE in Mongolia.

## Case presentation

**Figure 1:**
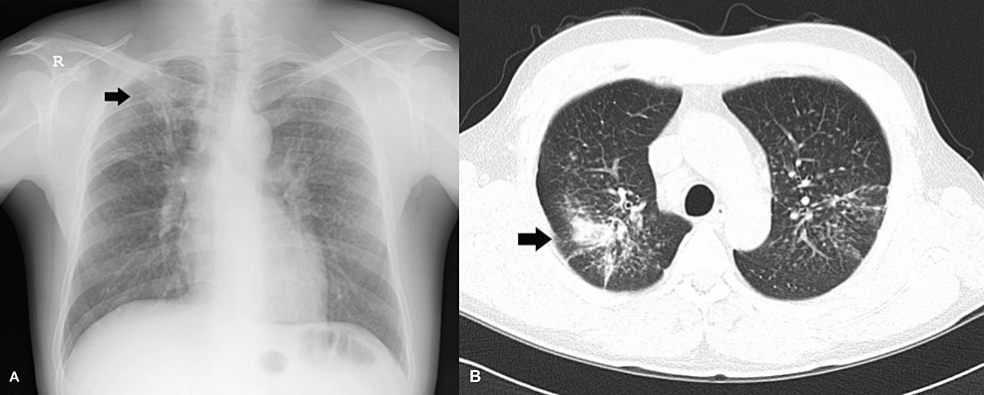
A. Chest radiograph shows multiple ill-defined small nodules in both lung fields, predominantly in the upper and middle zones with large opacities in the right upper lobe (black arrow). B. Axial thin-section computed tomography scan obtained multiple rounded centrilobular nodules in both lung fields with upper lobe dominance. There are conglomerated massive changes in the right upper lobe (black arrow) and coalescence of the small nodule in the left upper lobe.

A 39-year-old underground miner underwent chest radiography during a periodic medical check-up at the workplace. He had worked as a driller in the underground fluorspar mine for 20 years, except for a year of military service. Each shift lasted seven hours. The underground mine where the patient worked was often drilled dry, without water supplement or efficient personal protective equipment. He had a history of smoking half a pack of cigarettes daily for 15 years. Physical examination findings were unremarkable at diagnosis, and there were no abnormal sounds in both lung fields. Lung function tests showed a slightly restrictive pattern. The sputum test for pulmonary tuberculosis was negative. Chest radiography revealed ill-defined small, multiple nodular opacities of round shape presented with moderate contrast in all zones of both lungs and large opacity in the right upper zone. Chest computed tomography showed diffuse multiple rounded centrilobular nodules, with upper lobe dominance in both lung fields. Radiological examination revealed conglomerated massive changes in the right upper lobe, accompanied by the coalescence of small nodules in the left upper lung. Additionally, multiple enlarged lymph nodes were observed in the upper, lower paratracheal, and paraaortic regions (Figure 1).

**Figure 2:**
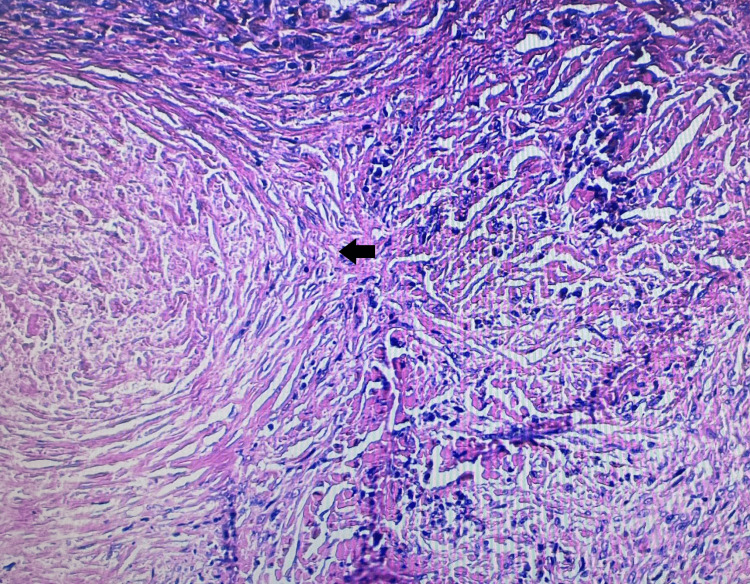
Lung biopsy shows silicotic nodule with concentric hyalinised collagen fibres surrounding an amorphous centre (black arrow), (hematoxylin-eosin ×10).

**Figure 3:**
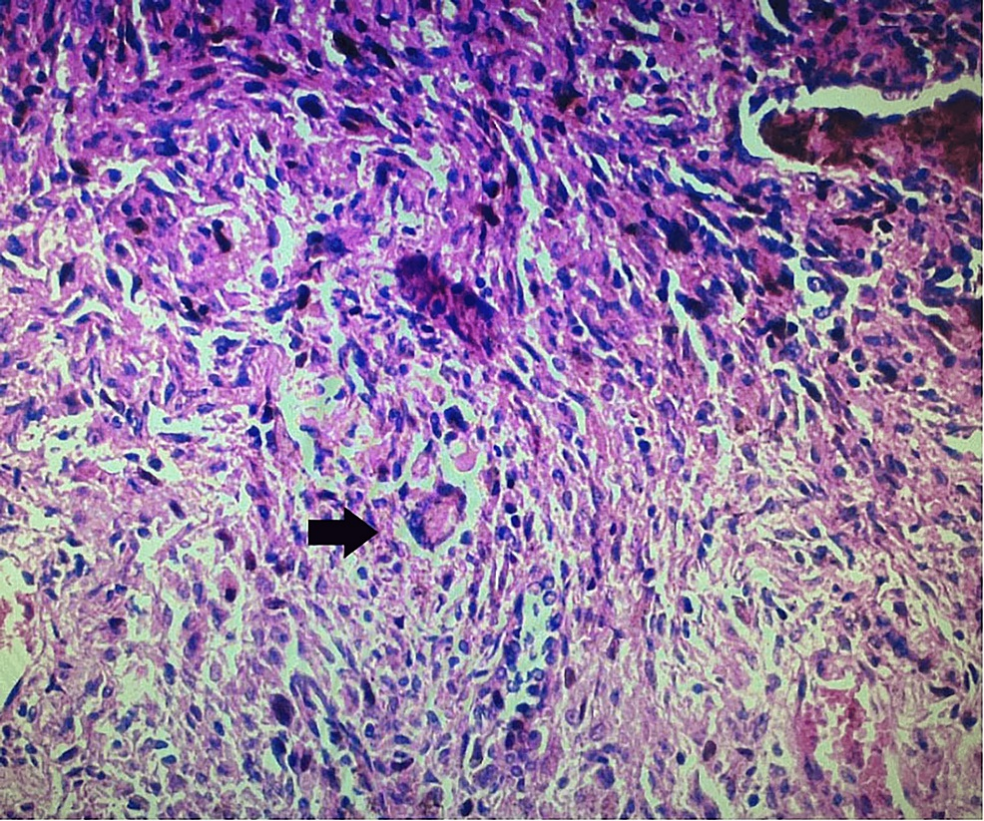
Lung biopsy shows sarcoidosis with Langshan’s giant cells surrounded by epithelioid cells and a few lymphocytic infiltrations (black arrow), (hematoxylin-eosin ×20).

Initially, the patient's work history and chest radiography suggested a diagnosis of silicosis. To rule out the possibility of a lung tumour and make a definitive diagnosis, additional blood tests were conducted to measure the levels of the following biomarkers: NSE, carcinoembryonic antigen (CEA), and cytokeratin 19 fragment (CYFRA 21-1). The blood level of the lung cancer marker NSE (24.58 ng/mL) was higher than normal (0-16.3). CEA (2.84 ng/mL; normal values 0-4.7) and CYFRA 21-1 (0.567 ng/mL; normal values 0-3.3) levels were normal. Hence, a tumour of the right upper lobe was suspected, and a right upper lobectomy was performed. The biopsy did not confirm a lung tumour. Histopathological examination of the lung specimen showed that the numerous silica nodules coalesced with each other, and there were symptoms associated with sarcoidosis. The features suggested two coexisting pathological processes: silicosis (Figure 2) and sarcoidosis (Figure 3).

**Figure 4:**
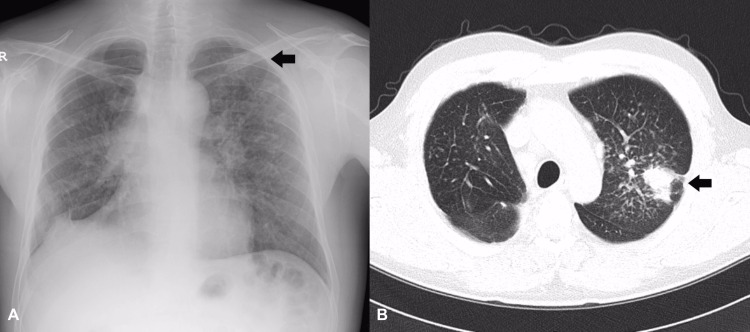
A. Numerous small nodular opacities involving both lungs, more in the left upper lung zone. Nodular opacities and focal pleural adhesion in the right hemidiaphragm. B. Lung resection state of the right upper lung, diffuse small bilateral nodules in both lung fields, with upper lobe dominance, and newly developed large fibrotic opacity in the left upper lobe.

Six months post-surgery, computed tomography showed a progressive massive fibrosis in the left upper lobe (Figure 4). The patient received methotrexate as a treatment for sarcoidosis. Four years post-surgery, the patient’s condition deteriorated as a result of lung tuberculosis, for which he received treatment.

Further, progressive massive fibrosis replaced both lung fields on the background of the reduced right lung field. Six years after the first diagnosis, the patient died from severe respiratory failure caused by silicosis, which had resulted in progressive massive lung fibrosis in both lungs.

## Discussion

Crystalline silica was considered a probable human carcinogen in 1987 by the International Agency for Research on Cancer (IARC). While silica itself has been debated as a carcinogen, research has highlighted that the risk of lung cancer is often linked to a complex interplay of factors. These include cigarette smoking, airflow obstruction, and exposure to radon daughters in underground mines. Notably, silica can also play a role in the carcinogenic process when combined with other substances, such as polycyclic aromatic hydrocarbons from cigarette smoke or industrial pyrolysis, which can act as carcinogens or promoters [14].

Tumour markers CEA, NSE, and CYFRA 21-1 are widely used for lung cancer screening and diagnosis [9-11,15]. This case demonstrated the combination of three clinical conditions diagnosed in one patient, including complicated silicosis associated with sarcoidosis and an elevated blood concentration of the NSE biomarker. The patient's work history and radiological symptoms were assessed to diagnose silicosis. Additional analysis of lung cancer biomarkers and elevation of serum NSE (24.58 ng/mL) led to lung cancer diagnosis, which was followed by right upper lobectomy surgery. The histological analysis showed the presence of silicosis nodules, Langshan’s cells, and epithelioid cells in our patient’s biopsy.

With an elevated level of serum NSE without an underlying neoplasm process, this case aligns with similar studies where serum tumour markers increased in non-malignant diseases [12,13,16-18]. Recent studies showed that elevated serum NSE might indicate silicosis, which helps to determine the early diagnosis and disease severity. One study analysed the changes in serum NSE levels in silicosis cases where serum NSE levels were significantly higher (22.88±7.86 ng/mL) in patients with silicosis than in healthy individuals (17.96±4.42 ng/mL) (p<0.05) [13]. Similar results were reported in a study that evaluated serum cancer antigen 125 (CA 125) and NSE biomarkers in the context of disease severity in patients with silicosis [17,18]. The results showed that serum NSE levels were significantly higher in silicosis cases than in non-silicosis cases. With the progression of silicosis, NSE levels increased gradually [17,18]. 

Some case reports and retrospective studies showed the relation between silica dust exposure and sarcoidosis [3,4,7,8]. Our patient had worked for many years in an underground mine as a driller, which might be a risk factor for sarcoidosis. A case series presented five cases of coexisting silicosis and sarcoidosis, showing that sequential associations of the two conditions can occur [7]. Computed tomography showed a rapid progression of silicosis in our patient within six months. The role of sarcoidosis in the deterioration of silicosis needs to be further discussed. The observed association between silica exposure and sarcoidosis suggests that sensitization to silica may play a role in the underlying immunological mechanisms, potentially contributing to the development of this condition [8].

Differentiating progressive massive fibrosis from lung cancer is clinically and radiologically important. In the early diagnosis and differential diagnosis of lung cancer, elevated blood levels of one tumour marker (NSE) possess lower sensitivity and specificity. Therefore, the combined detection of multiple markers can compensate for these defects. Magnetic resonance imaging might be useful for the differential diagnosis of progressive massive fibrosis and lung cancer. On magnetic resonance images, lung cancer appears as a high-signal-intensity lesion on T2-weighted images. Conversely, progressive massive fibrosis appears as a low-signal-intensity abnormality compared to the signal intensity of muscle on both T1- and T2-weighted images [19,20].

The diagnosis of silicosis requires a history of occupational exposure, compatible radiological features, and the exclusion of alternate diagnoses. The clinical management for silicosis and sarcoidosis differ. Silicosis is a non-curable yet preventable occupational lung disease, and sarcoidosis therapeutic interventions are available. Furthermore, patients with silicosis have an increased risk of infection with *Mycobacterium tuberculosis*, which was an additional factor in the exacerbation of our case. 

## Conclusions

In conclusion, this case highlights the potential utility of NSE as a biomarker for the early diagnosis and assessment of silicosis severity. Furthermore, it underscores the importance of a thorough differential diagnosis in cases where silicosis is suspected, particularly when coexisting lung cancer is a possibility, in order to ensure accurate and timely management.

## References

[1] Greenberg MI, Waksman J, Curtis J (2007). Silicosis: a review. Dis Mon.

[2] Hoy RF, Chambers DC (2020). Silica-related diseases in the modern world. Allergy.

[3] Syed T, Tiya S, Hemanth K, Muralimohan B (2014). Silicosis and sarcoidosis: a rare association. Chest J.

[4] Graff P, Larsson J, Bryngelsson IL, Wiebert P, Vihlborg P (2020). Sarcoidosis and silica dust exposure among men in Sweden: a case-control study. BMJ Open.

[5] Oliver LC, Zarnke AM (2021). Sarcoidosis: an occupational disease?. Chest.

[6] Oliver LC, Sampara P, Pearson D, Martell J, Zarnke AM (2022). Sarcoidosis in Northern Ontario hard-rock miners: A case series. Am J Ind Med.

[7] Yavuz MY, Demiral Y (2022). Coexistence of sarcoidosis and silicosis: case series. Respir Case Rep.

[8] Beijer E, Meek B, Kromhout H (2019). Sarcoidosis in a patient clinically diagnosed with silicosis; is silica associated sarcoidosis a new phenotype?. Respir Med Case Rep.

[9] Marangos PJ, Gazdar AF, Carney DN (1982). Neuron specific enolase in human small cell carcinoma cultures. Cancer Lett.

[10] Kaiser E, Kuzmits R, Pregant P, Burghuber O, Worofka W (1989). Clinical biochemistry of neuron specific enolase. Clin Chim Acta.

[11] Isgrò MA, Bottoni P, Scatena R (2015). Neuron-specific enolase as a biomarker: biochemical and clinical aspects. Adv Exp Med Biol.

[12] Bergamaschi S, Morato E, Bazzo M (2012). Tumor markers are elevated in patients with rheumatoid arthritis and do not indicate presence of cancer. Int J Rheum Dis.

[13] Bulut I, Arbak P, Coskun A, Balbay O, Annakkaya AN, Yavuz O, Gülcan E (2009). Comparison of serum CA 19.9, CA 125 and CEA levels with severity of chronic obstructive pulmonary disease. Med Princ Pract.

[14] Carta P, Aru G, Manca P (2001). Mortality from lung cancer among silicotic patients in Sardinia: an update study with 10 more years of follow up. Occup Environ Med.

[15] Schneider J (2010). Detection of lung cancer in silicosis patients using a tumor-marker panel. Cancer Biomark.

[16] Zhao YX, Dong ZP, Fan ZM, Shao HY, Cao QZ (2021). Changes of neuron specific enolase in serum of patients with silicosis [article in Chinese]. Zhonghua Lao Dong Wei Sheng Zhi Ye Bing Za Zhi.

[17] Fang SC, Zhang HT, Wang CY, Zhang YM (2014). Serum CA125 and NSE: biomarkers of disease severity in patients with silicosis. Clin Chim Acta.

[18] Huang HB, Huang JL, Xu XT, Huang KB, Lin YJ, Lin JB, Zhuang XB (2021). Serum neuron-specific enolase: A promising biomarker of silicosis. World J Clin Cases.

[19] Chong S, Lee KS, Chung MJ, Han J, Kwon OJ, Kim TS (2006). Pneumoconiosis: comparison of imaging and pathologic findings. Radiographics.

[20] Matsumoto S, Miyake H, Oga M, Takaki H, Mori H (1998). Diagnosis of lung cancer in a patient with pneumoconiosis and progressive massive fibrosis using MRI. Eur Radiol.

